# Silver-Russell syndrome secondary to rare (epi)genotypes exhibits phenotypic heterogeneity challenging clinical diagnosis

**DOI:** 10.1186/s13148-025-02023-7

**Published:** 2025-12-22

**Authors:** Uttara Kurup, David B. N. Lim, Avinaash V. Maharaj, Miho Ishida, Justin H. Davies, Helen L. Storr

**Affiliations:** 1 Centre for Endocrinology, William Harvey Research Institute (WHRI), John Vane Science Centre, Charterhouse Square, Barts and the London School of Medicine, London, EC1M 6BQ UK; 2 Paediatric Endocrinology, University Hospital Southampton NHS Foundation Trust, Southampton, SO16 6YD UK; 3 Faculty of Medicine, University of Southampton, Southampton, UK

**Keywords:** Silver-Russell syndrome, Diagnosis, Genetic, Monogenic, NH-CSS

## Abstract

**Context:**

Silver-Russell syndrome (SRS) is a complex multisystem condition requiring timely diagnosis for appropriate management. A clinical diagnosis is made in individuals scoring ≥ 4 Netchine-Harbison Clinical Scoring System (NH-CSS) criteria, with (epi)genetic investigations undertaken in those with NH-CSS ≥ 3 and strong clinical suspicion. Monogenic variants in imprinted (*CDKN1C* and *IGF2*) and non-imprinted (*HMGA2* and *PLAG1*) genes are recognised as rare causes of SRS. The frequency of associated phenotypes is unclear.

**Objective:**

We evaluated the suitability of SRS as an umbrella term for these (epi)genotypes by identifying key clinical features and assessing the validity of NH-CSS.

**Methods:**

An extensive literature search identified 22 *IGF2,* 18 *HMGA2*, 11 *CDKN1C* and 11 *PLAG1* published reports.

**Main outcome measure:**

Clinical phenotypes including the NH-CSS criteria were interrogated to assess (dis)similarity between the molecular subgroups of SRS.

**Results:**

Strict adherence to the NH-CSS identified clinical SRS in 91% *IGF2,* 82% *CDKN1C,* 78% *HMGA2* and 45% *PLAG1* affected individuals. Relative macrocephaly was observed in 82% *IGF2*, 82% *CDKN1C*, 44% *HMGA2*, and 27% *PLAG1* affected individuals. Prominent forehead was reported in 100% *CDKN1C*, 91% *IGF2*, 72% *HMGA2*, and 64% *PLAG1* and body asymmetry in 23% *IGF2* and 11% *HMGA2* affected individuals. Clinical features not typically associated with SRS included: microcephaly, challenging behaviour, cardiac abnormalities, cleft palate, and asthma.

**Conclusions:**

The NH-CSS missed 9–55% of monogenic SRS. The diverse phenotypes of *PLAG1, CDKN1C, HMGA2* and *IGF2* variants may hinder a clinical diagnosis of SRS. These rarer (epi)genotypes could be considered as distinct entities.

**Supplementary Information:**

The online version contains supplementary material available at 10.1186/s13148-025-02023-7.

## Introduction

Silver Russell syndrome (SRS, OMIM 180860) is a rare multisystem disorder characterised by pre- and postnatal growth failure with craniofacial dysmorphology. SRS is increasingly recognised as an important cause of short stature secondary to being born small for gestational age (SGA). Missed or delayed diagnosis is frequent since establishing the diagnosis is challenging and several conditions have overlapping phenotypes [1]. To facilitate the diagnosis, the Netchine-Harbison Clinical Scoring System (NH-CSS), based on a combination of characteristic features, is used due to its high sensitivity [2, 3].

The NH-CSS consists of 6 criteria: small for gestational age (SGA), postnatal growth failure, relative macrocephaly at birth, prominent forehead, body asymmetry, feeding difficulties and/or low body mass index (BMI) [1, 4]. ‘Associated features’ have been described in both SRS and non-SRS SGA short stature and are found in varying frequencies [4]. Additional features not typically observed in SRS have also been described and designated as ‘other features’ [1]. Individuals scoring at least four of six criteria, including prominent forehead and relative macrocephaly, are diagnosed as ‘Clinical SRS’. Molecular testing is recommended if three or more NH-CSS criteria are met, ultimately confirming the diagnosis in ~ 60% of individuals [4].

SRS is genetically heterogenous resulting from imprinted and non-imprinting abnormalities. Imprinting abnormalities include the common imprinted causes: loss of methylation of the *H19*/*IGF2* intergenic differentially methylated region (*H19*/*IGF2*:IG-DMR) at chromosome 11p15.5 (11p15LOM) and maternal uniparental disomy of chromosome 7 (upd(7)mat) accounting for 30–60% and 5–10% of affected individuals, respectively [5–7]. Rare monogenic causes of SRS account for approximately 5% and consist of variants in imprinted (*CDKN1C* [8] and *IGF2* [9]) and non-imprinted (*HMGA2* [10] and *PLAG1* [11]) genes.

Improving the recognition of SRS is crucial for prompt initiation of multidisciplinary management to achieve better patient outcomes. The NH-CSS demonstrates a high negative predictive value of 89% when identifying SRS secondary to 11p15LOM or upd(7)mat, however, its validity in identifying rarer causes of SRS is unknown. The phenotype of SRS secondary to rarer monogenic causes is not well characterised. The overarching objective of this study was to evaluate the effectiveness of the NH-CSS in identifying rare monogenic causes of SRS and collate the phenotype of these genotypes.

## Methods

The phenotypic spectrum of the ‘common imprinted causes’ of SRS (11p15LOM and upd(7)mat), were analysed using two published cohorts with individuals with SRS [2, 12]. The Azzi et al*.* cohort [2] assessed the NH-CSS criteria. The Wakeling et al*.* cohort [12], which employed the Price diagnostic scoring system [3], was used for all other comparisons.

A comprehensive literature review was undertaken to identify all published clinical reports of monogenic aetiologies (*CDKN1C, IGF2, HMGA2, PLAG1* gene variants) associated with SRS. All published reports of affected individuals with duplication or deletions of singular genes (monogenic) were included. Those affecting multiple genes were excluded. Duplicate reporting and affected individuals with incomplete phenotypic data were excluded from the cohort. All reports were reviewed by two authors independently and data extracted.

To understand the phenotypic differences between the six molecular subsets (11p15LOM, upd(7)mat, *CDKN1C*, *IGF2*, *HMGA2* and *PLAG1*) we analysed both grouped and individual molecular causes. Grouped molecular cause analyses included comparisons between:

i. Common imprinted causes (11p15LOM and upd(7)mat)
ii. Imprinted monogenic causes (*CDKN1C* and *IGF2*) and
iii. Non-imprinted monogenic causes (*HMGA2* and *PLAG1*)

We also compared individual monogenic causes (*CDKN1C*, *IGF2*, *HMGA2* and *PLAG1*) with 11p15LOM and upd(7)mat.

### Affected individuals and cohorts

#### Common imprinted (11p15LOM and upd(7)mat) SRS cohorts

Azzi reported 47 individuals with SRS, 35 (22 male 13 female) 11p15LOM and 12 upd(7)mat (7 male 5 female) (mean ages 6.9 years and 7.7 years, respectively). All individuals had detailed assessment using the NH-CSS and molecular confirmation of SRS [2].

Forty-four affected individuals with 11p15LOM (22 male 22 female) and 20 (5 male 15 female) with upd(7)mat (mean ages 6.3 years (range 0.8–26.8) and 7.2 years (range 1.3–17.9), respectively), were described in a prospective study by Wakeling [12]. All individuals with SRS had molecular confirmation following Price et al*.* scoring system assessment (birth weight ≤ 2 SD, preservation of occipitofrontal circumference, classical features, body asymmetry) [3]. SRS was confirmed if ≥ 4/5 Price scoring criteria were met [12].

#### Monogenic SRS cohorts (*CDKN1C*,* IGF2*,* HMGA2* and *PLAG1* gene variants)

##### *Monogenic imprinted gene (CDKN1C*,* IGF2) variants*

11 affected individuals with *CDKN1C* missense variants (mean age 10.9 years; range 1.1–21 years) [8, 13–18] and 22 (mean age 6.7; range 1.5–23 years) with *IGF2* truncating and missense variants were identified (**Supplementary Table 1a and b**) [1, 8, 9, 11, 15–33]. Individuals with *CDKN1C* variants comprised 2 males and 8 females and 1 individual with no sex documented. Of these, 4 were probands and 4 were family members from their extended kindreds and 3 were sporadic. The *IGF2* variant cohort comprised 14 males and 8 females. There were 2 probands with 4 family members and 16 sporadic variants.

#### Monogenic non-imprinted (HMGA2, PLAG1) gene variants

Eighteen affected individuals (10 females and 8 males, mean age 4.7 years; range 0.5–32 years) with *HMGA2* [10, 11, 34–41] and 11 (all females, mean age 3.08 years; range 0.1–9 years) with *PLAG1* variants were identified (**Supplementary Table 1a and b**) [1, 10, 11, 16, 24, 29–31, 34–47]. Individuals affected by *HMGA2* variants consisted of 2 probands, 3 extended kindreds and 14 sporadic variants. The *PLAG1* cohort consisted of 1 proband, 3 individuals from their extended kindreds and 7 sporadic variants.

### Netchine harbison clinical scoring system (NH-CSS) criteria analyses

To ascertain the usefulness of the NH-CSS criteria in the different molecular subgroups, we compared the frequency of common imprinted and monogenic causes which satisfied ≥ 4 NH-CSS criteria. For this analysis we only included published reports where all 6 criteria were documented *IGF2* (n = 22), *HMGA2* (n = 18), *CDKN1C* (n = 11), and *PLAG1* (n = 11). We then analysed the 6 individual features within the NH-CSS and compared the monogenic causes to the common imprinted causes.

The presence of major NH-CSS criteria i.e., relative macrocephaly (defined as head circumference at birth ≥ 1.5 SDS above birth weight and/or length SDS) and prominent forehead were analysed in more detail. This analysis only included reports from the cohorts above which had complete auxological data: *IGF2* (n = 19), *HMGA2* (n = 13), *CDKN1C* (n = 8), *PLAG1* (n = 7).

The frequency of microcephaly (% occipitofrontal circumference; OFC < -2 SDS) and normocephaly (% OFC within ± 2 SDS) was assessed in the different subgroups and segregated into relative microcephaly (% OFC > 1.5 SDS below birthweight SDS), relative normocephaly (% OFC within 1.5 SDS of birthweight SDS) and relative macrocephaly (% OFC > 1.5 SDS above birthweight SDS).

We compared birth weight SDS between the monogenic causes and common imprinted causes and OFC, birth length and height SDS between the monogenic causes of SRS. Height SDS was assessed at 24 months of age and at the latest clinical assessment.

### System involvement in common and monogenic causes of SRS

To understand system involvement by each genotype, clinical features were grouped into organ systems (neurological/neurodevelopmental, cardiology, gastroenterology, genitourinary, respiratory, endocrine, musculoskeletal, integumentary, and other) for analysis. We assessed system involvement by calculating the proportion of features attributed to a molecular cause within an organ system.

### Analysis of ‘associated’ and ‘other’ clinical features

The clinical features reported for all the molecular subtypes of SRS were collated and classified as ‘associated’ features if they were reported in but not specific to SRS [4] or ‘other features’ if not typically described in SRS [1]. All reported ‘associated’ and ‘other’ features were compared between the molecular causes (grouped and individual). The individual features were classified into antenatal (intrauterine growth, placental abnormalities) and postnatal (facial, musculoskeletal, dermatological, cardiological, neurological/neurodevelopmental, endocrine, gastroenterological and miscellaneous features) for comparison.

### Statistical analysis

Fisher exact and unpaired T-tests with two-sided p values were used, as appropriate, for statistical comparisons using GraphPad Prism (version 10.3.1, California, USA). P-values of < 0.05 were considered significant.

## Results

### Identification of individuals with SRS using NH-CSS stratified by (epi)genotype

**Fig. 1:**
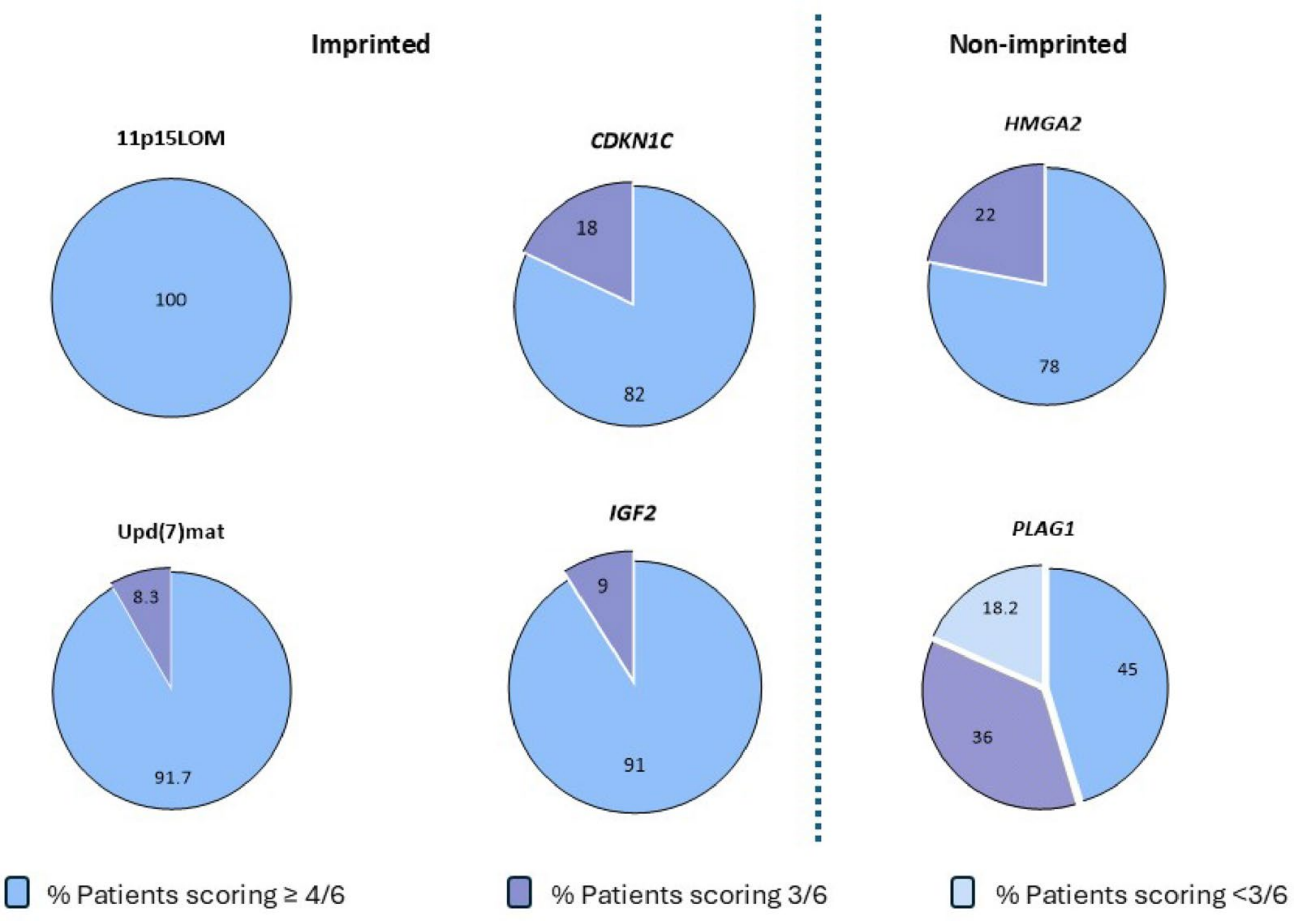
NH-CSS scores reported in individuals with imprinted (common and monogenic) and non-imprinted causes of SRS. NH-CSS compared between the molecular causes of SRS and categorised by ≥ 4/6 “Clinical SRS”, ≥ 3/6 requiring molecular investigation and < 3/6 denoting non-SRS causes. NH-CSS identified clinical SRS in 91% *IGF2,* 82% *CDKN1C,* 78% *HMGA2* and 45% *PLAG1* of affected individuals compared to 100% 11p15LOm and 91% upd(7)mat

Of the individuals with 11p15LOM (n = 35) and upd(7)mat (n = 12), 46/47 (98%) satisfied ≥ 4/6 NH-CSS criteria which identified 100% of individuals with 11p15LOM and 11/12 (92%) with upd(7)mat variants [2]. Strict adherence to NH-CSS identified monogenic SRS less frequently: 91% *IGF2, 82*% *CDKN1C,* 78% *HMGA2* and *45*% *PLAG1*. Moreover, 36% *PLAG1,* 22% *HMGA2*, 18% *CDKN1C* and 9% individuals with *IGF2* variants scored 3/6 NH-CSS compared to none of the 11p15LOM and 8.3% upd(7)mat [12]. Additionally, 18.2% of individuals with *PLAG1* exhibited < 3 NH-CSS (Fig. 1).

### Comparison of the individual NH-CSS criteria between common and monogenic SRS causes

#### Small for gestational age (SGA)

**Fig. 2:**
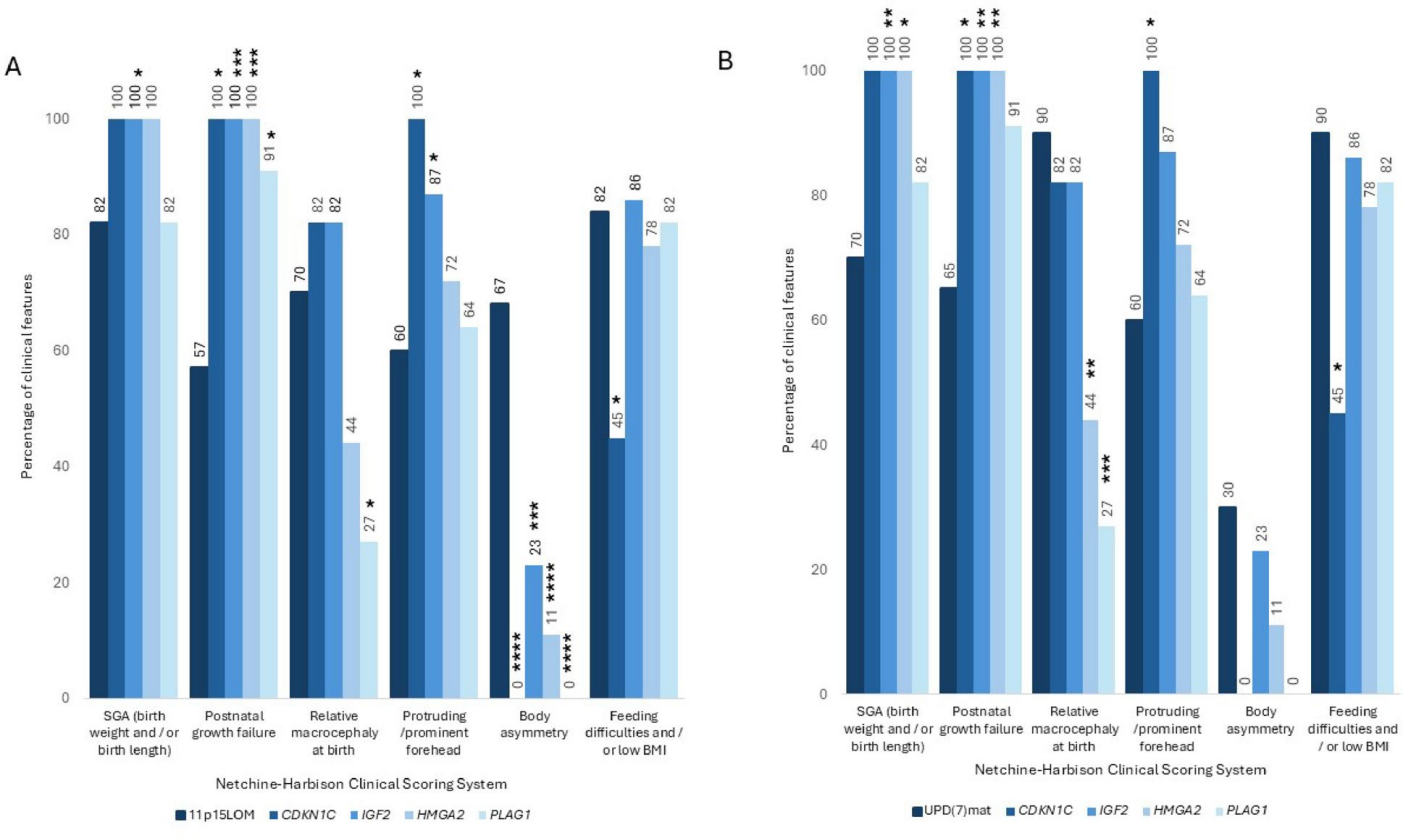
NH-CSS features in patients with 11p15 loss of methylation (11p15LOM) and maternal uniparental disomy (upd(7)mat) compared to monogenic causes of SRS. (**A**) Frequency of NH-CSS criteria were compared between patients with 11p15LOM and those with monogenic causes (**B**) Frequency of NH-CSS criteria were compared between patients with upd(7)mat and those with monogenic causes. Features showing statistically significant differences between the 11p15LOM (A) or Upd(7)mat (B) and respective monogenic cause of SRS are indicated as follows: *, *p* ≤ 0.05; **, *p* ≤ 0.01; ***, *p* ≤ 0.001; ****p ≤ 0.0001

SGA incidence was high in all molecular causes of SRS (82% 11p15LOM, 70% upd(7)mat, 100% *CDKN1C*, 100% *IGF2*, 100% *HMGA2* and 82% *PLAG1*) (Fig. 2a). Comparatively, SGA was significantly higher in: common imprinted vs imprinted monogenic causes (p = 0.002), *IGF2* vs 11p15LOM (p = 0.0445) and *IGF2* (p = 0.0064) and *HMGA2* (p = 0.0208) vs upd(7)mat (Figs. 2a and b and Supplementary Table 2)[31].

**Fig. 3:**
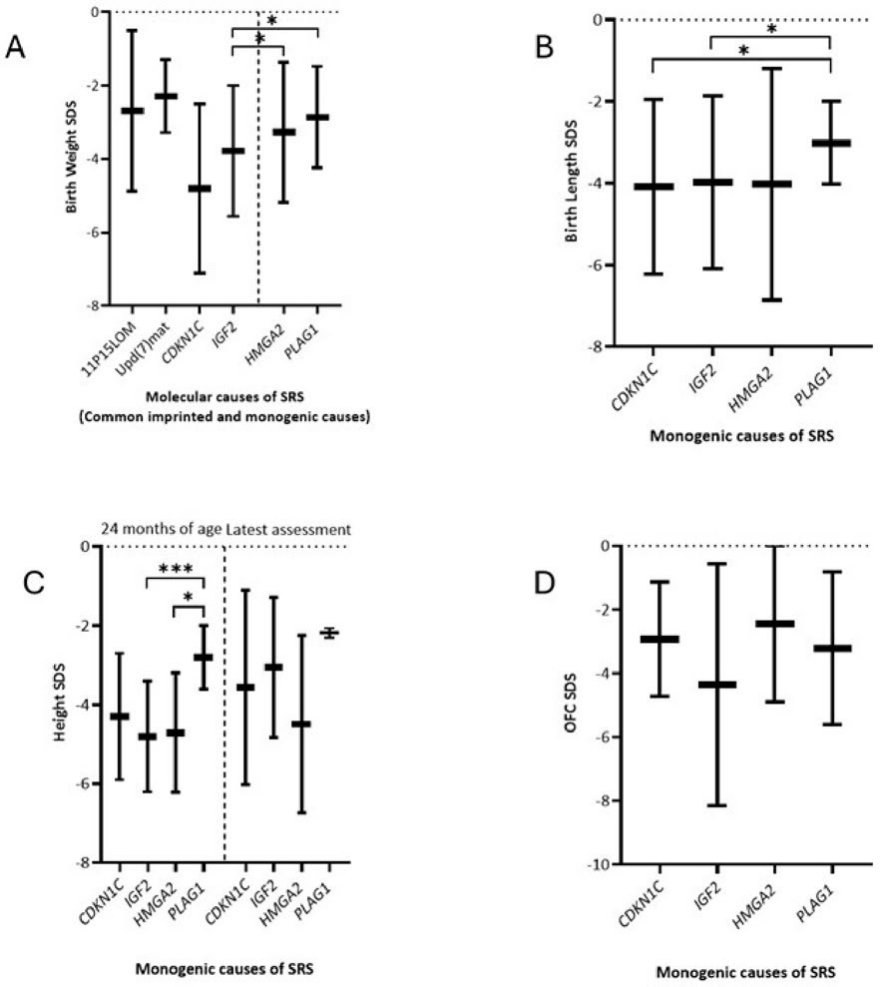
Comparison of birth weight, birth length, height and occipitofrontal circumference SDS between the molecular causes of SRS. Statistically significant differences in birth weight (**A**), birth length (**B**), height (**C**) and occipitofrontal circumference (**D**) SDS between the molecular causes of SRS are denoted by an asterisk (*) where: *, *p* ≤ 0.05; **, *p* ≤ 0.01; ***, *p* ≤ 0.001. No birth length, height or occipitofrontal SDS data range was available for 11p15LOM or upd(7)mat to make a comparison in Figs. 3b, c and d. All SDS values were derived from previously published reports

Of the monogenic causes, BW SDS was significantly lower in *IGF2* vs *HMGA2* and *PLAG1* (Fig. 3a). Birth lengths (BL) were not available for the common causes, but BL SDS were lower in *CDKN1C* and *IGF2* compared to *PLAG1* (Fig. 3a).

#### Postnatal growth failure (PNGF)

PNGF was the most prevalent NH-CSS feature in monogenic SRS (100% *CDKN1C, HMGA2* and *IGF2* and 91% *PLAG1*) (Figs. 2a and b). There was a significantly higher incidence in imprinted monogenic (97%) and non-imprinted monogenic (100%) compared to common imprinted causes (59%) (**Supplementary Tables 2 and 3**) but not significantly different between imprinted and non-imprinted monogenic causes (**Supplementary Table 4**) [31]. Frequency of PNGF was significantly higher in all individual monogenic causes than 11p15LOM (57%) and *CDKN1C, HMGA2* and *IGF2* compared to upd(7)mat (65%) (Fig. 2a and 2b). Height SDS at 24 months was significantly lower in *HMGA2* (*p* = 0.0312) and *IGF2* (*p* = 0.0009) compared to *PLAG1* (Fig. 3c).

#### Relative macrocephaly

Relative macrocephaly was commoner in common imprinted (90% upd(7)mat, 70% 11p15LOM) and imprinted monogenic (82% *CDKN1C* and *IGF2)* vs non-imprinted monogenic (44% *HMGA2,* 27% *PLAG1*) causes. The frequency of relative macrocephaly was significantly higher in 11p15LOM compared to *PLAG1* (27%) and and upd(7) mat compared to HMGA2(44%) and PLAG1(27%) (Figs. 2a and b).

Published literature demonstrated relative macrocephaly in 50% *PLAG1*, 45.4% *IGF2* and 28.5% *CDKN1C.* In contrast, none of the individuals with *HMGA2* variants presented with relative macrocephaly, whereas the other monogenic causes had microcephaly (87.5% *CDKN1C*, 57.8% *IGF2*, 57.1% *PLAG1* and 53.8% *HMGA2*). One individual with *IGF2* (9%) presented with relative microcephaly. Significantly more individuals with *HMGA2* (46.1%), *PLAG1* (42.8%) and *IGF2* (42.1%) variants presented with normocephaly at birth compared to *CDKN1C* (12.5%). Relative normocephaly was present in 100% *HMGA2*, 71.4% *CDKN1C*, 50% *PLAG1* and 45.4% *IGF2* (Table 1). Average OFC SDS was lowest in *IGF2* but there was no statistical difference between monogenic causes (Fig. 3d).

**Table 1:** Occipitofrontal circumference and prominent forehead comparison between monogenic molecular causes of SRS

Molecular causes (n*)	Mean OFC SDS (range)	Relative macrocephaly % (n) (range)	Normo-cephaly	Relative normocephaly % (n) (range)	Microcephaly % (n) (range)	Relative microcephaly % (n) (range)	% Prominent forehead
*CDKN1C* (n = 8)	– 2.86 (– 1.12 to – 4.73)	29 (2/7) (– 2.14 to – 4.73)	13 (1/8) (– 1.12)	71 (5/7) (– 2.1 to – 4.2)	88 (7/8) (– 2.14 to – 4.73)	0	100 (8/8)
*IGF2* (n = 19)	– 2.14 (– 0.56 to – 8.14)	45.4 (5/11) (– 2 to – 2.6)	42 (8/19) (– 0.56 to – 1.5)	45 (5/11) (– 2.07 to – 3.5)	58 (11/19) (– 2 to – 8.14)	9 (1/11) (– 8.14)	95 (18/19)
*HMGA2* (n = 13)	– 2.1 (+ 0 to – 4.9)	0	46 (6/13) (– 1.97 to + 0)	100 (7/7) (– 2 to – 4.9)	54 (7/13) (– 2 to – 4.9)	0	92 (12/13)
*PLAG1* (n = 7)	– 2.61 (– 0.81 to – 5.61)	50 (2/4) (– 3.45 to – 3.84)	43 (3/7) (– 0.81 to – 1.5)	50 (2/4) (– 2.2 to – 5.61)	57 (4/7) (– 2.2 to – 5.61)	0	71 (5/7)

n*, patients with complete auxological data available. Prominent forehead, forehead projecting beyond the facial plane on a side view as a toddler[1–3 years]); microcephaly, % Occipitofrontal circumference below – 2 SDS; relative macrocephaly, % Occipitofrontal circumference SDS more than 1.5 SDS above birthweight SDS; relative normocephaly, % Occipitofrontal circumference SDS within 1.5 SDS of birthweight SDS; relative microcephaly, % Occipitofrontal circumference SDS more than 1.5 SDS below birthweight SDS

#### Prominent forehead

Prominent forehead was reported equally (60%) in 11p15LOM and upd(7)mat but was present in a higher proportion of monogenic causes (100% *CDKN1C*, 87% *IGF2*, 72% *HMGA2* and 64% *PLAG1*) (Figs. 2a and b). Prominent forehead was more common in imprinted monogenic causes (94%) compared to common imprinted (59%) and non-imprinted monogenic causes (69%) (**Supplementary Tables 2, 3 and 4)**[31].

Prominent forehead was significantly greater in all imprinted monogenic causes (100% *CDKN1C* and 87% *IGF2*) vs 11p15LOM (60%) (Fig. 2a). When monogenic causes were compared to upd(7)mat (60%), only *CDKN1C* (100%) reached significance (Fig. 2b).

#### Body asymmetry

Body asymmetry was the least common NH-CSS feature amongst monogenic causes (23% *IGF2*, 11% *HMGA2* and absent in *CDKN1C* and *PLAG1*) (Figs. 2a and b). Asymmetry was significantly higher in common imprinted causes (56%) than imprinted monogenic causes (15%) and non-imprinted monogenic causes (7%) (**Supplementary Tables 2, 3 and 4**)[31]. It was significantly higher in 11p15LOM (67%) than any monogenic cause (Fig. 2a). Asymmetry had a similar incidence in upd(7)mat (30%) when compared to the monogenic causes (Fig. 2b).

#### Feeding difficulties and/or low BMI

The incidence of feeding difficulties and/or low BMI was comparable between 11p15LOM (82%), upd(7)mat (90%), *IGF2* (86%), *PLAG1* (82%), *HMGA2* (78%)and *CDKN1C* (45%) (Figs. 2a and b) and no significant differences were found in grouped (**Supplementary Tables 2, 3 and 4**) or individual analyses except *CDKN1C* which presented with a lower frequency [31].

### System involvement in common and monogenic causes of SRS

SRS is a multisystem disorder. Interestingly, there was relative absence of respiratory system involvement in all molecular causes except *CDKN1C*, where asthma was reported in 18%. 11p15LOM had features across all other systems. The absence of cardiac features in upd(7)mat, *CDKN1C*, *HMGA2* and *PLAG1* demarcated these causes from 11p15LOM and *IGF2*. In addition, *IGF2* and *CDKN1C* lacked gastroenterological abnormalities. Musculoskeletal system abnormalities were most consistent amongst all causes of SRS. *PLAG1* exhibited the least extensive multisystem involvement, with features primarily restricted to the neurodevelopmental/neurological, gastroenterological, endocrinological, and musculoskeletal systems.

### Comparison of ‘associated’ and ‘other’ features between molecular causes of SRS

We compared ‘associated’ and ‘other’ features of SRS (**Supplementary Tables 5, 6, 7, 8 and 9)** between the different molecular causes.

#### Antenatal features

Placental abnormalities (including placental hypoplasia) were higher in common imprinted compared to non-imprinted monogenic causes (**Supplementary Tables 5, 6 and 7**) [31]. 11p15LOM (34%) had a higher frequency of placental abnormalities than *CDKN1C* (0%) and *HMGA2* (6%) (**Supplementary Table 8**) [31]. The presence of placental abnormalities in upd(7)mat (10%) was not significantly different when compared to monogenic causes (**Supplementary Table 9**) [31].

#### Facial features

Abnormal dentition, micro/retrognathia, downturned mouth, delayed fontanelle closure and abnormalities of the lip were more frequent in common imprinted causes compared to imprinted monogenic causes (**Supplementary Table 5**) [31]. Additionally, abnormalities of the ear were more common in non-imprinted monogenic causes compared to common imprinted causes (**Supplementary Table 6) **[31]. The presence of cleft palate (18%) in imprinted monogenic causes was significantly higher compared to non-imprinted monogenic causes (**Supplementary Table 7**) [31]. Notably, the presence of cleft palate was significantly higher in *IGF2* (27%) compared to upd(7)mat (0%) (**Supplementary Table 9**) [31].

#### Musculoskeletal features

Multiple skeletal abnormalities were reported in monogenic causes, including polydactyly, clinodactyly, syndactyly, camptodactyly, genu valgum and elbow pterygium. Congenital limb abnormalities in common imprinted causes of SRS were predominantly in the upper extremities [12]. Fifth finger clinodactyly was significantly higher in common imprinted causes (66%) when compared to non-imprinted monogenic causes (24%) (**Supplementary Table 3) **[31].

#### Dermatological features

The presence of pigmented naevi was significantly higher for *IGF2* variants (23%) both when compared to 11p15LOM (0% *p* = 0.0029) and upd(7)mat (0%, *p* = 0.0492).

None of the rare monogenic causes reported excessive sweating as a clinical feature. 67% of the common imprinted causes combined (64% 11p15LOM and 75% upd(7)mat) reported excessive sweating which was significantly higher than in imprinted monogenic causes (0%) (**Supplementary Table 2**) and non-imprinted causes (0%) (**Supplementary Table 3**) [31].

#### Cardiac features

The presence of congenital cardiac abnormalities was higher in imprinted monogenic causes (27%), but not non-imprinted causes, when compared to common imprinted causes (6%) (**Supplementary Tables 2 and 3) **[31]. The presence of cardiac abnormalities was significantly higher in imprinted monogenic causes when compared to non-imprinted causes (0%) (**Supplementary Table 4**) [31].

The presence of cardiac abnormalities was significantly higher in *IGF2* (41%) compared to 11p15LOM (9%) (**Supplementary Table 8**) [31]. The most common cardiac defects in *IGF2* were ventricular septal defect (44.4%, 4/9) and atrial septal defect (22.2%, 2/9). Patent ductus arteriosus, bicuspid aortic arch and total anomalous pulmonary venous return (TAPVR) were also reported in monogenic causes.

#### Neurological and neurodevelopmental features

Common imprinted causes had lower frequency of motor delay and higher frequency of speech delay compared to imprinted monogenic and non-imprinted monogenic causes respectively (**Supplementary Tables 2 and 3**) [31]. Intellectual disability/learning disabilities were present in low frequencies in *IGF2* (5%) while behavioural abnormalities were reported in 11p15LOM (9%), upd(7)mat (20%) and *IGF2* (5%).

#### Endocrine features

The presence of hypoglycaemia was higher in common imprinted causes (26%) when compared to imprinted monogenic causes (0%) and non-imprinted monogenic causes (**Supplementary Tables 2 and 3**) [31].

#### Gastroenterological features

The presence of gastroesophageal reflux was higher in 11p15LOM (14%) and upd(7)mat (10%) compared to *HMGA2* (11%).

#### ENT

The presence of a high-pitched voice was significantly higher in *IGF2* (23%) compared to common imprinted causes (11p15LOM (0%) and upd(7)mat (0%).

## Discussion

Diagnosing SRS remains a unique challenge due to the diminishing prominence of features with age and phenotypic overlap with several syndromes [1, 48]. Different scoring systems have been proposed to improve SRS identification [3, 6, 49] but the international consensus statement recommended the NH-CSS as the optimal clinical tool to identify SRS [4]. Making a clinical diagnosis of SRS can be challenging as several conditions have overlapping features and may fulfil the clinical diagnostic criteria of the NH-CSS. The use of WES and/or appropriate gene panels may address this issue [1, 15]. Over the past decade, and since publication of the consensus statement, the molecular spectrum of SRS has expanded to include rare monogenic causes (*CDKN1C, IGF2, HMGA2, PLAG1*) in addition to the commoner causes (11p15LOM and upd(7)mat). The phenotype of these rarer genotypes has not been studied in detail and although the NH-CSS is sensitive for identifying common imprinted causes of SRS, it has not been validated for monogenic causes. Our findings show that the NH-CSS has low sensitivity for identifying individuals with *CDKN1C, IGF2, HMGA2* and *PLAG1* variants*,* and that these genotypes have a much more diverse phenotype than SRS secondary to 11p15LOM or upd(7)mat*.* A high proportion of monogenic causes do not fulfil the required four or more clinical NH-CSS features; 54% *PLAG1*, 22% *HMGA2*, 18% *CDKN1C* and 9% *IGF2* would have been missed. Another study also found variable phenotypes in some rare monogenic causes of SRS [50].

Monogenic causes of SRS have lower mean birth weight SDS than the common causes, but low sample size makes it difficult to assess the significance. As SGA can be defined by birth weight or length < -2 SDS, it is possible that birth length SDS may be a better marker of SGA than birth weight SDS for monogenic causes of SRS. However, these auxological data are not universally recorded. The higher frequency of postnatal growth failure was a prominent feature of monogenic causes while the low frequency of body asymmetry was evident in monogenic cause of SRS. Our analysis of height SDS demonstrated that failure of linear growth secondary to monogenic causes persists into later life. The involvement of major NH-CSS clinical criteria (prominent forehead and relative macrocephaly) was inconsistent. While relative macrocephaly was frequently absent in non-imprinted causes (*HMGA2* and *PLAG1*), the frequency of prominent forehead was significantly higher in imprinted monogenic causes (*CDKN1C* and *IGF2*).

Relative macrocephaly (defined as head circumference > 1.5 SDS above birth weight and/or length) is common in 11p15LOM and upd(7)mat. We found that only *CDKN1C*, *IGF2* and *PLAG1* demonstrated true relative macrocephaly. However, head sparing (normocephaly) was seen in 13% *CDKN1C*, 43% *PLAG1*, 42% *IGF2* and 46% *HMGA2*. All monogenic causes had individuals with microcephaly (88% *CDKN1C*, 58% *IGF2*, 57% *PLAG1* and 54% *HMGA2)*. This has previously been reported for *HMGA2* and *PLAG1* [44, 45], only one *IGF2* case had relative microcephaly. This demonstrates the need to further stratify occipitofrontal circumference into relative and absolute values to understand neurocortical and allometric growth effects of these genes relative to body size(29) [51,52]. Feeding difficulties were consistently demonstrated in both common and monogenic causes.

SRS is a multisystemic disorder, with growth restriction beginning in utero. Monogenic causes restrict growth via inhibition of cell proliferation (*CDKN1C*) [8] or by direct or indirect *IGF2* reduction (*IGF2* imprinted gene or *HMGA2-PLAG1-IGF2* pathway)(8,16,23,45). It is possible that the diverse phenotypes observed between genetic causes are due to differences in *IGF2* dysregulation [53]. Interestingly, this correlates with growth restriction in the presence of placental abnormalities in SRS secondary to monogenic causes. Overall musculoskeletal disorders (including craniofacial dysmorphology) and integumentary problems remain the most frequent findings in SRS. Monogenic disorders, specifically *IGF2*, presented with a range of ‘associated’ and ‘other’ features. For example, asthma and challenging behaviour in *CDKN1C*, triangular facies, cleft palate, ectrodactyly, presence of pigmented nevi, high-pitched voice and male genital abnormalities in *IGF2*, microcephaly in *HMGA2*, and microcephaly, learning difficulties and thin hair in *PLAG1*. Congenital cardiac disorders were largely restricted to 11p15LOM and *IGF2* variants. Interestingly, clinical features of cases with *IGF2* variants exhibit a propensity for midline structural defects exhibiting cleft and cardiac abnormalities. Poor cardiometabolic health has been studied in SRS(38) [54], but not in monogenic causes.

Although *CDKN1C, PLAG1, HMGA2* and *IGF2* phenotypes can overlap with SRS secondary to 11p15LOM or upd(7)mat, this is inconsistent. With the rarer genotypes, key features such as relative macrocephaly, needed to make a clinical diagnosis of SRS using NH-CSS, are often absent, and there is a high frequency of features not typically observed in SRS due to 11p15LOM and upd(7)mat.

We recognise the limitations of our study. It relies solely on published manuscripts to identify phenotypic differences between molecular subtypes of SRS. This introduces the possibility that certain phenotypic features were overlooked, unreported, or genuinely absent. Additionally, published reports may disproportionately represent more severe phenotypes, potentially limiting the generalisability of findings to milder presentations. We acknowledge the limitations of conducting statistical analyses on small sample sizes and that statistical comparisons across phenotypic groups may increase the risk of false-positive findings. However, these issues are common to descriptions of all ultra-rare disorders and difficult to resolve.

The phenotypes of *CDKN1C, PLAG1, HMGA2* and *IGF2* are likely too diverse to be considered SRS. We, therefore, recommend first line molecular investigation for 11p15 LOM and upd(7)mat for individuals scoring ≥ 3/6 NH-CSS and that a diagnosis of SRS is reserved just for these epigenotypes. It would be pertinent to undertake second-line testing for monogenic causes of SRS in those individuals negative for 11p15 LOM and upd(7)mat and scoring ≥ 3/6 NH-CSS or with high clinical suspicion of SRS. *CDKN1C, PLAG1, HMGA2* and *IGF2* variants remain important causes of SGA and post-natal growth failure, and these genes should be included in short stature gene panels, even if no features of SRS are present. The fundamental management of SRS remains a multidisciplinary approach led by paediatric endocrinologists. Early growth hormone therapy can improve growth/final height, regardless of the molecular background, and discourses on diagnostic terminology must not divert from this [55]. Further study of the natural history of *CDKN1C, PLAG1, HMGA2* and *IGF2* variants is needed to inform management strategies as these genotypes may require different approaches from those used in 11p15LOM and upd(7)mat.

## Supplementary Information

Below is the link to the electronic supplementary material.

Supplementary Material 1

Supplementary Material 2

## Data Availability

All the original data generated and analysed during this study are included in this manuscript. Supplemental data are available in Mendeley Data, 10.17632/mbbwf2wb49.1
