# Supplementary material for: Silver-Russell syndrome secondary to rare (epi)genotypes exhibits phenotypic heterogeneity challenging clinical diagnosis
Source: Clin Epigenetics. 2025 Dec 22;17:208. doi: 10.1186/s13148-025-02023-7 (PMC12723925; doi:10.1186/s13148-025-02023-7)
Supplement: Supplementary file 1 — Supplementary Material 1 [file 13148_2025_2023_MOESM1_ESM.docx]

**Supplemental Table 1a Phenotypes of reported genetic variants: NH-CSS and other associated clinical features.** Adapted from [1]

| **Pt** | | **Variant** | **Inheritance** | | **NH-CSS score** | | **Other associated SRS features** | | **Other features** | | **Refs** | |  |
| --- | --- | --- | --- | --- | --- | --- | --- | --- | --- | --- | --- | --- | --- |
| Individuals with ***CDKN1C*** gene variants reported in literature | | | | | | | | | | | | | |
| 1 | | NM_000076.2  c.835C>A  p.Arg279Ser  Missense  Het  Likely pathogenic: PM2, PM1, PM5, PP3, PP4, PP1 | Mat | | 5/6 a,b,c,d,f | | NA | | NA | | [2] | |  |
| 2 | | NM_000076.2  c.835C>A  p.Arg279Ser  Missense  Het  Likely pathogenic: PM2, PM1, PM5, PP3, PP4, PP1 | Mat | | 5/6 a,b,c,d,f | | Triangular face  Male genital abnormalities  Motor delay | | Prematurity  Oligohydramnios | | [2] | |  |
| 3 | | NM_000076.2  c.836G>T p.Arg279Leu  Missense  Het  Pathogenic: PM1, PM2, PS1, PP3, PP1_strong, PP4 | Mat | | 3/6 a,b,d | | Triangular face  Clinodactyly | | Short stature  Otitis media with effusion  Retro-orbital and temporal headaches  Hypermetropia  Recurrent nose bleeds  Small ears  High anterior hairline  Mid facial crowding  High arched palate  Incurving of the lateral aspect of the toenails  Joint laxity (Beighton 7/9)  IGF-I slightly above normal (68.7 nmol/L) thus IGF-I resistance | | [3] | |  |
| 4 | | NM_000076.2  c.836G>T p.Arg279Leu  Missense  Het Male    Pathogenic: PM1, PM2, PS1, PP3, PP1_strong, PP4 | Mat | | 3/6 a,b,d | | NA | | Short stature  Delay in tooth eruption  Asthma in childhood  Poor concentration  Challenging behaviour  Dyspraxia  Asperger’s  High anterior hairline  Small hands and feet  Poor response to hGH therapy | | [3] | |  |
| 5 | | NM_000076.2  c.836G>T  p.Arg279Leu  Missense  Het  Pathogenic: PP1_strong, PS3, PM1, PM2, PP3, PP4 | Mat | | 5/6 a,b,c,d,f | | Triangular face | | NA | | [4] | |  |
| 6 | | NM_000076.2  c.836G>T p.Arg279Leu  Missense  Het  Pathogenic: PP1_strong, PS3, PM1, PM2, PP3, PP4 | Mat | | 4/6 a,b,c,d | | Triangular face | | NA | | [4] | |  |
| 7 | | NM_000076.2  c.836G>T p.Arg279Leu  Missense  Het  Pathogenic: PP1_strong, PS3, PM1, PM2, PP3, PP4 | Mat | | 4/6 a,b,c,d | | Triangular face | | IUGR | | [4] | |  |
| 8 | | NM_000076.2  c.836G>T  p.Arg279Leu  Missense  Het  Pathogenic: PP1_strong, PS3, PM1, PM2, PP3, PP4 | Mat | | 5/6 a,b,c,d,f | | Triangular face | | NA | | [4] | |  |
| 9 | | NM_001122631.1  c.809G>C  p.Arg281Thr (NM_001122630.2; p.Arg270Thr)  Missense  Het  Likely pathognic: PM2, PM1, PP3, PP4 | Mat | | 5/6 a,b,c,d,f | | NA | | IUGR  Oligohydramnios  Short stature | | [5] | |  |
| 10 | | NM_000076.2  c.947G>A  p.Arg316Gln  Missense  Het  Likely pathogenic: PS3, PM2, PP3, PP4 | Mat | | 4/6 a,b,c,d | | Triangular face | | Left inguinal hernia  Cafe au lait spots  Congenital dermal melanocytosis  Asthma  Dysmenorrhea | | [6] | |  |
| 11 | | NM_000076.2  c.836G>A  p.Arg279His  Missense  Pathogenic: PP1_strong, PM2, PM5, PM1, PP3, PP4 | Mat | | 4/6 a,b,c,d | | Triangular face  Low set ears  Delayed closure of fontanelle  Irregular crowded teeth | | Prematurity  Sunken eyes | | [7] | |  |
| Individuals with ***IGF2*** gene variants reported in literature | | | | | | | | | | | | | |
| 12 | | NM_000612.5   c.78C> G  p.Tyr26*  Premature stop  Het  Pathogenic: PVS1, PS2, PM2 | De novo | | 5/6 a,b,c,d,f | | Triangular face | | NA | | [8] | |  |
| 13 | | NM_000612.5  c.158_159dup  p.Arg54Alafs*7  Premature stop  Het  Pathogenic: PVS1, PS2, PM2 | De novo | | 5/6 a,b,c,d,f | | Triangular face | | NA | | [8] | |  |
| 14 | | NM_001291861.3  c.443C>T  p.Ala148Val  Missense  Het  VUS: PM2, BP4 | Pat | | 3/6 a,b,c | | Clinodactyly | | IUGR  Intellectual disability  Small placenta  Cafe au lait spots  Short stature | | [5] | |  |
| 15 | | NM_001127598.2  c.191C>A  p.Ser64Ter  Loss-of-function  Nonsense  Pathogenic: PVS1, PP1_Strong, PM2, PP4 | Pat | | 6/6 a,b,c,d,e,f | | Triangular face  Clinodactyly  Low set ears  High pitched voice  Delayed closure fontanelle  Male genitalia abnormalities (hypospadias)  Speech delay  Motor delay  Syndactyly | | PDA  VSD  Pigmented nevi  Preauricular pits / fistulae  Reduced stamina  Ectrodactyly  Delayed puberty  Ray defect of the right ulna  Elbow pterygium  Delayed bone age  Oligohydramnios | | [9] | |  |
| 16 | | NM_001127598.2  c.191C>A  p.Ser64Ter  Loss-of-function  Nonsense  Pathogenic: PVS1, PP1_Strong, PM2, PP4 | Pat | | 5/6 a,b,c,d,f | | Triangular face  Clinodactyly  Low set ears  High pitched voice  Speech delay  Motor delay  Syndactyly | | PDA  Pigmented nevi  Preauricular pits / fistulae  Reduced stamina  Delayed bone age  Prematurity | | [9] | |  |
| 17 | | NM_001127598.2  c.191C>A  p.Ser64Ter  Loss-of-function  Nonsense  Pathogenic: PVS1, PP1_Strong, PM2, PP4 | Pat | | 5/6 a,b,c,d,f | | Triangular face  Clinodactyly  Low set ears  High pitched voice  Male genitalia abnormalities (hypospadias and cryptorchidism)  Motor delay | | Pigmented nevi  Preauricular pits / fistulae  Reduced stamina  Hypercholesterolemia  Delayed bone age  Prematurity | | [9] | |  |
| 18 | | NM_001127598.2  c.191C>A  p.Ser64Ter  Loss-of-function  Nonsense  Pathogenic: PVS1, PP1_Strong, PM2, PP4 | Pat | | 5/6 a,b,c,d,f | | Triangular face  Clinodactyly  Low muscle mass  Low set ears  High pitched voice  Motor delay  Syndactyly | | VSD  Pigmented nevi  Preauricular pits / fistulae  Prematurity | | [9] | |  |
| 19 | | NM_000612.6  c.–6–1G>C  Exon 2 skipping  Canonical splice site  Pathogenic: PVS1, PS2, PM2, PP4 | De novo | | 5/6 a,b,c,d,f | | Triangular face  Clinodactyly  Micrognathia  Speech delay  Motor delay | | TAPVR  Placental hypoplasia  Cleft palate | | [10] | |  |
| 20 | | NM_000612.6  c.209G>A  p.Cys70Tyr  Missense  Pathogenic: PS2, PP3, PM2, PM1, PP4 | De novo | | 5/6 a,b,c,d,f | | Triangular face  Clinodactyly  Micrognathia  Male genitalia abnormalities (hypospadias and bifid scrotum)  Speech delay  Motor delay | | Cleft palate  Hypoplastic fingernails  Placental hypoplasia | | [6,10] | |  |
| 21 | | NM_000612.6  c.211T>C  p.Cys71Arg  Missense  Likely pathogenic: PP3, PM2, PM1, PM6, PP4 | Pat/De novo | | 6/6 a,b,c,d,e,f | | Triangular face  Clinodactyly  Micrognathia  Male genitalia abnormalities (hypospadias, abnormal scrotum)  Speech delay  Motor delay | | Cleft palate  Placental hypoplasia  Oligohydramnios | | [6,10] | |  |
| 22 | | NM_000612.6  c.97T>A  p.Cys33Ser  Missense  Pathogenic: PS2, PM2, PM5, PP3, PM1, PP4 | De novo | | 5/6 a,b,c,d,f | | Triangular face  Clinodactyly  Micrognathia | | Delayed puberty  Ectrodactyly  Cervical vertebral fusion  Cleft palate | | [10] | |  |
| 23 | | NM_000612.6  c.134G>C  p.Cys45Ser  Missense  Pathogenic: PS2, PP3, PM1, PM2, PP4 | De novo | | 3/6 a,b,f | | Speech delay  Motor delay | | Precocious puberty | | [10] | |  |
| 24 | | NM_000612  c.101G>A  p.Gly34Asp  Het    Pathogenic: PS2, PM2, PM5, PP3, PM1,  PP4 | De novo | | 5/6 a,b,c,d,e | | Triangular face  Clinodactyly  Micrognathia  Lowe set ears  High pitched voice  Male genitalia abnormalities (microphallus and hydrocele)  Irregular teeth | | ASD  Reduced stamina  Pigmented nevi | | [11] | |  |
| 25 | | NM_000612.5  c.110_117delinsAGGTAA p.Leu37Glnfs*31  Frameshift    Pathogenic: PVS1, PS2, PM2, PP4 | De novo | | 5/6 a,b,c,d,f | | Triangular face  Clinodactyly  Micrognathia  Low set ears  Male genitalia abnormalities (microphallus and hydrocele)  Speech delay  Motor delay  Syndactyly | | Cleft palate  Polydactyly  Ectrodactyly  Hearing impairment  Pulmonary hypertension  Respiratory distress syndrome | | [12] | |  |
| 26 | | NM_000612.5  c.157+3A>C  Splice site  Het  Pathogenic: PS3, PS2, PM2, PP4 | De novo | | 5/6 a,b,c,d,f | | Triangular face  Shoulder dimples  Micrognathia  Speech delay  Motor delay | | VSD  PVL | | [13] | |  |
| 27 | | NM_000612  c.195delC  p.Ile66Serfs*93  Het  Pathogenic: PVS1, PS2, PM2, PP4 | De novo | | 5/6 a,b,c,d,f | | Triangular face  Clinodactyly  Micrognathia  Low set ears  Male genitalia abnormalities (abnormal scrotum)  Speech delay  Motor delay | | PDA  Cleft palate | | [14] | |  |
| 28 | | NM_001127598   c.381T>G  p.Cys127Trp (NM_000612.6: p.Cys71Trp)  Het  Likely pathogenic: PM1, PM2, PM5, PP3, PP4 | Pat | | 5/6 a,b,d,e,f | | Triangular face  Downturned mouth | | Bicuspid aortic valve with mild stenosis  Deep set eyes  Depressed nasal bridge  Long philtrum  Delayed bone age | | [15] | |  |
| 29 | | NM_001127598   c.381T>G  p.Cys127Trp (NM_000612.6: p.Cys71Trp)  Het  Likely pathogenic: PM1, PM2, PM5, PP3, PP4 | Pat | | 4/6 a,d,e,f | | Triangular face  Clinodactyly  Micrognathia  Downturned mouth | | VSD  ASD  Bicuspid aortic valve  Smooth philtrum | | [15] | |  |
| 30 | | NM_000612.5  c.122T>G p.Leu41Arg  Missense  Het  Likely pathogenic: PS2, PP3, PM2, PP4 | De novo | | 5/6 a,b,c,d,f | | Triangular face  Clinodactyly  Speech delay  Motor delay | | NA | | [16] | |  |
| 31 | | NM_000612.4  c.157+5G>A  Splice site  Het    Pathogenic: PS3, PS2, PM2, PP4 | De novo | | 5/6 a,b,c,d,f | | Triangular face  Clinodactyly  Micrognathia  Low muscle mass  Low set ears  Speech delay  Motor delay | | Small placenta  Delayed bone age  Oligohydramnios | | [17] | |  |
| 32 | | NM_000612  c.[-6-2A>G]  Het splice variant    Pathogenic: PVS1, PM2, PP4 | Pat | | 4/6a,b,c,f | |  | |  | | [18] | |  |
| 33 | | NM_000612.6  c.518dupC P.Glu174ArgfsTer50  Het  VUS: PM4, PM2_supporting, PP3, PP4 | Pat | | 4/6a,b,c,d | |  | | Prominent ears  Long eyelashes  Midface hypoplasia  Slightly elongated filtrum  Microstomy  Thin lips | | [19] | |  |
| Individuals with ***HMGA2*** gene variants reported in literature | | | | | | | | | | | | | |
| 34 | | NM_003483.4  c.166A>G  p.Lys56Glu  Missense  Het    Pathogenic: PS3, PM1, PM2, PP3, PP4 | Mat | | 3/6 ^a,b,f^ | | Triangular face  High-pitched voice  Motor delay | | IUGR  High-arched palate  Precocious puberty  Mild developmental delay (inattention and poor writing/reading skills)  Microcephaly (OFC -4.9 SDS) | | [20] | |  |
| 35 | | NM_003483.4  c.145delA, p.R49Gfs*117  Frameshift  Het    Pathogenic: PVS1, PM2, PP4 | Unknown | | 3/6 ^a,b,c^ | | NA | | IUGR  Puffy hands and feet at birth | | [20] | |  |
| 36 | | NM_003483.4  c.52C>T  p.Q18*  Nonsense  Het  Pathogenic: PVS1, PM2, PP4 | Mat | | 4/6 ^a,b,d,e^ | | Triangular face | | IUGR  Microcephaly (OFC -2.0 SDS)  Hypermetropia  Astigmatism | | [20] | |  |
| 37 | | NM_003483.4  c.144delC, p.R49Gfs*117  Frameshift  Het  Pathogenic: PVS1, PM2, PP4 | Unknown | | 4/6 ^a,b,d,f^ | | High-pitched voice | | GORD  Mid-facial hypoplasia | | [20] | |  |
| 38 | | NM_003483.4  c.49G>T, p.G17*  Nonsense  Het  Pathogenic: PVS1, PM2, PP4 | Mat | | 3/6 ^a,b,f^ | | NA | | NA | | [20] | |  |
| 39 | | NM_003483.4  c.193C>T  p.Gln65*  Premature stop Deleterious  Het  Pathogenic: PVS1, PM2, PP4 | De novo | | 5/6 ^a,b,c,d,f^ | | Triangular face | | NA | | [8] | |  |
| 40 | | NM_003483.4  c.189del  p.Ala64Leufs*102  Frameshift  Deleterious  Het  Pathogenic: PVS1, PM2, PP4 | Unknown | | 5/6 ^a,b,c,d,f^ | | Triangular face | | NA | | [8] | |  |
| 41 | | NM_003483. 4  7bp intron 4 del  Het  Pathogenic: PVS1, PM2, PP1_supportive, PP4 | Mat | | 5/6 ^a,b,c,d,f^ | | Triangular face  Clinodactyly  Micrognathia  Low muscle mass  Syndactyly | | IUGR  GORD  Café au lait spot  Epicanthus  Hypertelorism  Clitoral hypertrophy  Thin upper lip | | [21] | |  |
| 42 | | NM_003483.4  Del exon 1 to 3  Het  Pathogenic: PVS1, PM2, PP4 | Pat | | 4/6 ^a,b,d,f^ | | Triangular face | | IUGR | | [22] | |  |
| 43 | | NM_003483.4  Del exon 1 to 3  Het  Pathogenic: PVS1, PM2, PP4 | Pat | | 4/6 ^a,b,c,d^ | | NA | | Type 2 diabetes as an adult | | [22] | |  |
| 44 | | NM_003483. 4  c.111 + 1G>T,  p.?  Pathogenic: PVS1, PM2, PP4 | De novo | | 4/6 ^a,b,d,f^ | | Triangular face  Abnormal teeth | | Cafe au lait spots  Genu valgum  Microcephaly (OFC -3.31 SDS) | | [22] | |  |
| 45 | | NM_003483. 4  c.239C>T,  p.Pro80Leu  Missense  Hom  Pathogenic: PP1_strong, PM1, PM2, PP3, PP4 | Mat / Pat | | 4/6 ^a,b,d,f^      *Macrocephaly and body asymmetry unknown* | | Triangular face  Clinodactyly | | Delayed teething  Deep set eyes  Small nose and ears  Craniofacial disproportion  Narrow palate | | [22] | |  |
| 46 | | NM_003483. 4  c.239C>T  p.Pro80Leu  Missense  Hom  Pathogenic: PP1_strong, PM1, PM2, PP3, PP4 | Mat / Pat | | 4/6 ^a,b,d,f^    *Macrocephaly and body asymmetry unknown* | | Triangular face  Clinodactyly | | Midface hypoplasia  Small nose and ears  Craniofacial disproportion  Narrow palate | | [22] | |  |
| 47 | | NM_003483.4  12q14.3  7.3kb deletion of exon 1 and 2  Het  Pathogenic: PVS1, PS2, PM2, PP4 | De novo | | 4/6 ^a,b,d,f^ | | Triangular face | | Microcephaly (OFC -2.0 SDS)  Delayed bone maturation | | [23] | |  |
| 48 | | NM_003483.6  c.27dup  p.Gln10Alafs*33  Pathogenic: PVS1, PM250, PP4 | Paternal | | 4/6^a,b,c,f^ | | Typical facies | |  | | [24] | |  |
| 49 | | c.111 + 5G > A  VUS: PS2, PM2, PP3 | De novo | | 3/6^a,b,e^ | | Triangular face  micrognathia | | Hypertelorism  long eyelashes  delayed bone age | | [25] | |  |
| 50 | | 13 Kb-deletion (including 3 exons)  Het  Pathogenic: PVS1, PM2, PP4 | Maternal | | 5/6^a,b,c,d,f^ | | Triangular facies  retromicrognathia   clinodactyly  joint hypermobility | |  | | [26] | |  |
| 51 | | NM_003483.6  c.138_141delinsCT  p. Lys46Asnfs*16  Het  Pathogenic:, PVS1, PS2, PM2, PP4 | De novo | | 5/6 ^a,b,c,d,f^ | | triangular face  micrognathia  fifth finger clinodactyly | | Oligohydramnios  almond-shaped palpebral fissure  concave nasal ridge  small hands | | [27] | |  |
| Individuals with ***PLAG1*** gene variants reported in literature | | | | | | | | | | | | | |
| 52 | NM_002655.2  c.439delA  p.Ser147Valfs*82  Premature stop  Het  Pathogenic:, PVS1, PM2, PP4 | | | Mat | | 3/6 ^a,b,d^ | | Triangular face | | NA | [8] |  |  |
| 53 | NM_002655.2  c.439delA  p.Ser147Valfs*82  Premature stop  Het  Pathogenic:, PVS1, PM2, PP4 | | | Mat | | 3/6 ^a,b,f^ | | Triangular face | | NA | [8] |  |  |
| 54 | NM_002655.2  c.439delA  p.Ser147Valfs*82  Premature stop  Het  Pathogenic:, PVS1, PM2, PP4 | | | Mat | | 4/6 ^a,b,d,f^ | | Triangular face | | NA | [8] |  |  |
| 55 | NM_002655.2  c.1363delC  p.Gln455Serfs*16  Premature stop  Het  Pathogenic: PM2, PVS1, PP4, PS2 | | | De novo | | 5/6 ^a,b,c,d,f^ | | Triangular face | | NA | [8] |  |  |
| 56 | NM_002655.2  c.551delA p.Lys184Serfs*45  Het    Pathogenic: PVS1, PM2, PP4 | | | Mat | | 3/6 ^a,b,f^ | | Triangular face  Speech delay  Crowded teeth  Motor delay | | IUGR  Microcephaly (OFC -5.6 SDS)  Gestational hypertension  Gestational diabetes  Small placenta  Subtle superficial haemangioma in the frontal midline  Thin hair  Long forehead  Bulbous small nose  Prominent chin  High arched palate  Fine voice | [28] |  |  |
| 57 | NM_002655.2  c.599dup  p.Arg-201Profs*52  Frameshift  Pathogenic: PVS1, PM2, PP4, PM6 | | | Unknown/De novo | | 4/6 ^a,b,d,f^ | | Triangular face  Micrognathia | | NA | [15] |  |  |
| 58 | NM_002655.3  c.589C>T  p.Arg197*  Nonsense  Pathogenic:, PVS1, PM2, PP4 | | | Mat | | 5/6 ^a,b,c,d,f^ | | Triangular face  Clinodactyly | | ADHD  Hypothyroidism (after GH therapy) | [6] |  |  |
| 59 | NM_002655.2  c.1023del  p.Tyr341*  Loss-of-function  Likely pathogenic: PVS1, PM2, PP4 | | | Unknown | | 3/6 ^b,d,f^ | | Clinodactyly  Speech delay | | IUGR  Microcephaly (OFC <-2 SDS)  Parietal bossing  Short stature  Malnutrition  Vomiting | [29] |  |  |
| 60 | NM_002655.2  c.688C>T  p.Arg230*  Loss-of-function    Likely pathogenic: PVS1, PM2 | | | Unknown | | 2/6 | | Motor delay | | IUGR  Microcephaly (OFC <-2 SDS)  Abnormal hip joint morphology and instability  Protruding ear and abnormality of earlobe  Right face prominence  Thin upper lip vermilion  Short palpebral fissure | [29] |  |  |
| 61 | NM_002655.3: c.131del; p.Asn44Thrfs*6 Het  Pathogenic: PVS1, PM2, PP4 | | | Pat | | 4/6^a,b,c,d^ | | Triangular face | |  | [30] |  |  |
| 62 | NM_002655.3: c.402delT p.Gly135Aspfs*94  Het  Likely pathogenic: PVS1, PM2 | | | Mat | | 2/6^a,f^ | |  | | Short long bone | [31] |  |  |

Literature search identified individuals with *CDKN1C* variants (n=11), *IGF2* variants (n=22), *HMGA2* variants (n=18) and *PLAG1* variants (n=11) for which Netchine-Harbison clinical scoring system and any other clinical features were analysed and described. Corresponding variants and ACMG[32,33] categories are described. Pt, reported participant , Het, Heterozygous gene defect; Mat, Maternal; IUGR, intrauterine growth restriction; IGF-I, insulin-like growth factor 1; hGH, growth hormone therapy; PDA, persistent ductus arteriosus; VSD, ventricular septal defect; ASD, atrial septal defect; TAPVR, total anomalous pulmonary venous return; PVL, post ventricular white matter loss and periventricular leukomalacia; GORD, gastro-oesophageal reflux disease; ADHD, Attention Deficit Hyperactivity Disorder; SDS, standard deviation; NA, not available.

**Supplementary Table 1b. List of published variants excluded from analysis due to incomplete phenotypic data.**

| **Pt** | **Variant** | **Refs** |
| --- | --- | --- |
| **Excluded *CDKN1C* variants** | | |
| 1. | NM_000076.2 Chromosome 11p15 c.836G>[G;T], Participant reference: II.1 | [4] |
| 2. | NM_000076.2 Chromosome 11p15 c.836G>A, Participant reference: III.2 | [7] |
| 3. | NM_000076.2 Chromosome 11p15 c.836G>A, Participant reference: II.3 | [7] |
| 4. | NM_000076.2 Chromosome 11p15 c.836G>A, Participant reference: II.1 | [7] |
| 5. | NM_000076.2 Chromosome 11p15 c.836G>A, Participant reference: I.1 | [7] |
| 6. | NM_000076.2 Chromosome 11p15 c.842G>T, multiple family members described | [34] |
| **Excluded *IGF2* variants** | | |
| 7. | NM_001291861.3 c.245C>T, Participant reference: 64 | [5] |
| 8. | NM_001127598.1 c.267C>A, Participant reference: 6009 | [35] |
| **Excluded *HMGA2* variants** | | |
| 9. | 12q14 microdeletion (8.95Mb), Participant reference: #D0801870 | [36] |
| 10. | 7bp deletion microdeletion eliminated the 3′ AG acceptor site of intron 4, Participant reference: affected mother | [21] |
| 11. | NM_003483.4 c.223C>T, Participant reference: 14 | [37] |
| 12. | ENST00000536545 c.223C>T, Participant reference: 19 | [38] |
| **Excluded *PLAG1* variants** | | |
| 13. | NM_002655.3 c.551delA, Participant reference: III.4 | [28] |
| 14. | NM_002655.3 c.131del, Participant reference: Proband’s mother | [30] |
| 15. | NM_002655.3 c.402delT, Participant reference: Proband’s mother | [31] |

 Participant reference: Identifier used in the respective publications. 6 *CDKN1C,* 2 *IGF2*, 4 *HMGA2* and 3 *PLAG1* variants were excluded due to insufficient phenotypic data.

​​**References**

1. Kurup U, Lim DBN, Palau H, Maharaj A V, Ishida M, Davies JH, et al. Approach to the Patient With Suspected Silver-Russell Syndrome. J Clin Endocrinol Metab. 2024;

2. Binder G, Ziegler J, Schweizer R, Habhab W, Haack TB, Heinrich T, et al. Novel mutation points to a hot spot in CDKN1C causing Silver-Russell syndrome. Clin Epigenetics. 2020;12:152.

3. Sabir AH, Ryan G, Mohammed Z, Kirk J, Kiely N, Thyagarajan M, et al. Familial Russell–Silver Syndrome like Phenotype in the PCNA Domain of the *CDKN1C* Gene, a Further Case. Case Rep Genet. 2019;2019:1–8.

4. Brioude F, Oliver-Petit I, Blaise A, Praz F, Rossignol S, Le Jule M, et al. CDKN1C mutation affecting the PCNA-binding domain as a cause of familial Russell Silver syndrome. J Med Genet. 2013;50:823–30.

5. Alhendi ASN, Lim D, McKee S, McEntagart M, Tatton-Brown K, Temple IK, et al. Whole-genome analysis as a diagnostic tool for patients referred for diagnosis of Silver-Russell syndrome: a real-world study. J Med Genet. 2022;59:613–22.

6. Inoue T, Nakamura A, Iwahashi-Odano M, Tanase-Nakao K, Matsubara K, Nishioka J, et al. Contribution of gene mutations to Silver-Russell syndrome phenotype: multigene sequencing analysis in 92 etiology-unknown patients. Clin Epigenetics. 2020;12:86.

7. Li J, Chen L-N, He H-L. CDKN1C gene mutation causing familial Silver-Russell syndrome: A case report and review of literature. World J Clin Cases. 2023;11:4655–63.

8. Abi Habib W, Brioude F, Edouard T, Bennett JT, Lienhardt-Roussie A, Tixier F, et al. Genetic disruption of the oncogenic HMGA2-PLAG1-IGF2 pathway causes fetal growth restriction. Genet Med. 2018;20:250–8.

9. Begemann M, Zirn B, Santen G, Wirthgen E, Soellner L, Büttel H-M, et al. Paternally Inherited IGF2 Mutation and Growth Restriction. New England Journal of Medicine. 2015;373:349–56.

10. Masunaga Y, Inoue T, Yamoto K, Fujisawa Y, Sato Y, Kawashima-Sonoyama Y, et al. IGF2 Mutations. J Clin Endocrinol Metab. 2020;105.

11. Liu D, Wang Y, Yang X-A, Liu D. De Novo Mutation of Paternal IGF2 Gene Causing Silver–Russell Syndrome in a Sporadic Patient. Front Genet. 2017;8.

12. Yamoto K, Saitsu H, Nakagawa N, Nakajima H, Hasegawa T, Fujisawa Y, et al. De novo IGF2 mutation on the paternal allele in a patient with Silver–Russell syndrome and ectrodactyly. Hum Mutat. 2017;38:953–8.

13. Poulton C, Azmanov D, Atkinson V, Beilby J, Ewans L, Gration D, et al. Silver Russel syndrome in an aboriginal patient from Australia. Am J Med Genet A. 2018;176:2561–3.

14. Rockstroh D, Pfäffle H, Le Duc D, Rößler F, Schlensog-Schuster F, Heiker JT, et al. A new p.(Ile66Serfs*93) IGF2 variant is associated with pre- and postnatal growth retardation. Eur J Endocrinol. 2019;180:K1–13.

15. Meyer R, Begemann M, Hübner CT, Dey D, Kuechler A, Elgizouli M, et al. One test for all: whole exome sequencing significantly improves the diagnostic yield in growth retarded patients referred for molecular testing for Silver–Russell syndrome. Orphanet J Rare Dis. 2021;16:42.

16. Loid P, Lipsanen-Nyman M, Ala-Mello S, Hannula-Jouppi K, Kere J, Mäkitie O, et al. Case report: A novel de novo IGF2 missense variant in a Finnish patient with Silver-Russell syndrome. Front Pediatr. 2022;10:969881.

17. Xia C-L, Lyu Y, Li C, Li H, Zhang Z-T, Yin S-W, et al. Rare De Novo IGF2 Variant on the Paternal Allele in a Patient With Silver–Russell Syndrome. Front Genet. 2019;10.

18. Ventresca S, Lepri FR, Criscuolo S, Bottaro G, Novelli A, Loche S, et al. Case report: Long term response to growth hormone in a child with Silver-Russell syndrome-like phenotype due to a novel paternally inherited IGF2 variant. Front Endocrinol (Lausanne). 2024;15.

19. Özdemir BS, Sezer A, Erdeve ŞS, Çetinkaya S. A Novel IGF2 Gene Variant of Paternal Origin Causing the Silver-Russell Syndrome Phenotype. Horm Res Paediatr. 2023;

20. Maharaj A V., Cottrell E, Thanasupawat T, Joustra SD, Triggs-Raine B, Fujimoto M, et al. Characterization of HMGA2 variants expands the spectrum of Silver-Russell syndrome. JCI Insight. 2024;9.

21. De Crescenzo A, Citro V, Freschi A, Sparago A, Palumbo O, Cubellis MV, et al. A splicing mutation of the HMGA2 gene is associated with Silver–Russell syndrome phenotype. J Hum Genet. 2015;60:287–93.

22. Hübner CT, Meyer R, Kenawy A, Ambrozaityte L, Matuleviciene A, Kraft F, et al. HMGA2 Variants in Silver-Russell Syndrome: Homozygous and Heterozygous Occurrence. J Clin Endocrinol Metab. 2020;105:2401–7.

23. Leszinski GS, Warncke K, Hoefele J, Wagner M. A case report and review of the literature indicate that HMGA2 should be added as a disease gene for Silver-Russell syndrome. Gene. 2018;663:110–4.

24. Fisch-Shvalb N, Shefer-Averbuch N. A novel heterozygous pathogenic variant in the HMGA2 gene causing Silver-Russell Syndrome, a case-report. Horm Res Paediatr. 2023;

25. Bourousis E, Xatzipsalti M, Polychroni I, Kanavakis E, Stamoyannou L. A variant of uncertain significance of the HMGA2 gene in a child with Silver-Russell syndrome-like phenotype: a case report. Hormones. 2024;23:591–3.

26. Keselman A, Sanguineti N, Scaglia P, Casali B, Braslavsky D, Azcoiti ME, et al. Familial Silver-Russell like Syndrome with postnatal microcephaly caused by a partial HMGA2 gene deletion. Hormone Research in Paediatrics . 2023;

27. Yamoto K, Saitsu H, Ohkubo Y, Kagami M, Ogata T. Pathogenic sequence variant and microdeletion affecting HMGA2 in Silver–Russell syndrome: case reports and literature review. Clin Epigenetics. 2024;16:73.

28. Vado Y, Pereda A, Llano-Rivas I, Gorria-Redondo N, Díez I, Perez de Nanclares G. Novel Variant in PLAG1 in a Familial Case with Silver-Russell Syndrome Suspicion. Genes (Basel). 2020;11.

29. Chen W, Gruber A, Kyriss M, Bend E, Sullivan R, Keppler-Noreuil K. P145: Microcephaly in atypical Silver-Russell syndrome caused by defects in PLAG1. Genetics in Medicine Open. 2023;1:100174.

30. Dong P, Zhang N, Zhang Y, Liu C, Li C. Clinical characterization of PLAG1- related Silver-Russell syndrome：A clinical report. Eur J Med Genet. 2023;66:104837.

31. Tse WT, Bass C, Gurney L, Kinning E. Maternally inherited autosomal dominant PLAG‐1 related Silver Russell syndrome in a fetus with intra‐uterine growth restriction. Prenat Diagn. 2023;43:724–6.

32. Durkie M, Cassidy E-J, Berry I, Owens M, Turnbull C, Scott RH, et al. ACGS Best Practice Guidelines for Variant Classification in Rare Disease 2023 Recommendations ratified by ACGS Quality Subcommittee on xxx [Internet]. Royal Devon University Healthcare NHS Foundation Trust. 2023. Available from: https://www.cangene-canvaruk.org/gene-specific-recommendations.

33. Richards S, Aziz N, Bale S, Bick D, Das S, Gastier-Foster J, et al. Standards and guidelines for the interpretation of sequence variants: a joint consensus recommendation of the American College of Medical Genetics and Genomics and the Association for Molecular Pathology. Genetics in Medicine. 2015;17:405–24.

34. Kerns SL, Guevara-Aguirre J, Andrew S, Geng J, Guevara C, Guevara-Aguirre M, et al. A novel variant in CDKN1C is associated with intrauterine growth restriction, short stature, and early-adulthood-onset diabetes. J Clin Endocrinol Metab. 2014;99:E2117-22.

35. James KN, Clark MM, Camp B, Kint C, Schols P, Batalov S, et al. Partially automated whole-genome sequencing reanalysis of previously undiagnosed pediatric patients can efficiently yield new diagnoses. NPJ Genom Med. 2020;5:33.

36. Buysse K, Reardon W, Mehta L, Costa T, Fagerstrom C, Kingsbury DJ, et al. The 12q14 microdeletion syndrome: Additional patients and further evidence that HMGA2 is an important genetic determinant for human height. Eur J Med Genet. 2009;52:101–7.

37. Plachy L, Strakova V, Elblova L, Obermannova B, Kolouskova S, Snajderova M, et al. High Prevalence of Growth Plate Gene Variants in Children With Familial Short Stature Treated With GH. J Clin Endocrinol Metab. 2019;104:4273–81.

38. Vishnopolska SA, Mercogliano MF, Camilletti MA, Mortensen AH, Braslavsky D, Keselman A, et al. Comprehensive Identification of Pathogenic Gene Variants in Patients With Neuroendocrine Disorders. J Clin Endocrinol Metab. 2021;106:1956–76.
