# Supplementary material for: Silver-Russell syndrome secondary to rare (epi)genotypes exhibits phenotypic heterogeneity challenging clinical diagnosis
Source: Clin Epigenetics. 2025 Dec 22;17:208. doi: 10.1186/s13148-025-02023-7 (PMC12723925; doi:10.1186/s13148-025-02023-7)
Supplement: Supplementary file 2 — Supplementary Material 2 [file 13148_2025_2023_MOESM2_ESM.docx]

**SUPPLEMENTARY TABLES**

**Supplemental Table 2** Comparison of NH-CSS features in patients with common (11p15LOM and upd(7)mat) and monogenic imprinted causes (*CDKN1C* and *IGF2*) of SRS

| **Number of clinical features (%)** | **Common imprinted** | **Imprinted monogenic** | **P-value** |
| --- | --- | --- | --- |
| SGA | 50(78) | 33(100) | 0.0020 |
| Postnatal growth  failure | 38(59) | 33(100) | <0.0001 |
| Relative  macrocephaly | 48(75) | 27(82) | 0.60 |
| Prominent forehead | 38(59) | 31(94) | 0.0003 |
| Body asymmetry | 36(56) | 5(15) | <0.0001 |
| Feeding difficulty &/or low BMI | 54(84) | 24(73) | 0.18 |

*NH-CSS major criteria [1, 2. Results are presented as % (number of patients).

**Supplemental Table 3** Comparison of NH-CSS features in patients with common imprinted causes (11p15LOM and upd(7)mat) and non-imprinted monogenic causes (*HMGA2* and *PLAG1*) of SRS*.*

| **Number of clinical features (%)** | **Common imprinted** | **Imprinted monogenic** | **P-value** |
| --- | --- | --- | --- |
| SGA | 50(78) | 33(100) | 0.0020 |
| Postnatal growth  failure | 38(59) | 33(100) | <0.0001 |
| Relative  macrocephaly | 48(75) | 27(82) | 0.60 |
| Prominent forehead | 38(59) | 31(94) | 0.0003 |
| Body asymmetry | 36(56) | 5(15) | <0.0001 |
| Feeding difficulty &/or low BMI | 54(84) | 24(73) | 0.18 |

*NH-CSS major criteria 1, 2. Results are presented as % (number of patients).

**Supplemental Table 4** Comparison of NH-CSS features in patients with imprinted monogenic causes (*CDKN1C* and *IGF2*) and non-imprinted monogenic causes (*HMGA2* and *PLAG1*) of SRS*.*

| **Number of clinical features (%)** | **Imprinted monogenic** | **Non-imprinted monogenic** | **P-value** |
| --- | --- | --- | --- |
| SGA | 33(100) | 27(93) | 0.21 |
| Postnatal growth  failure | 33(100) | 28(97) | 0.46 |
| Relative  macrocephaly | 27(82) | 11(38) | 0.0006 |
| Prominent forehead | 31(94) | 20(69) | 0.0174 |
| Body asymmetry | 5(15) | 2(7) | 0.43 |
| Feeding difficulty &/or low BMI | 24(73) | 23(79) | 0.57 |

*NH-CSS major criteria 1, 2. Results are presented as % (number of patients).

**Supplemental Table 5** Associated and other features in Silver-Russell Syndrome patients with common imprinted causes compared to imprinted monogenic causes

| **Number of clinical features (%)** | **Common imprinted**  **Total n = 64** | **Imprinted monogenic causes**  **Total n = 33** | **p-value** |
| --- | --- | --- | --- |
| Placental abnormalities | 17(27) | 5(15) | 0.30 |
| Abnormal dentition | 34(53) | 3(9) | <0.0001 |
| Micro/retrognathia | 35(55) | 10(30) | 0.0314 |
| Downturned mouth | 17(27) | 2(6) | 0.0161 |
| Delayed closure of fontanelle | 16(41) | 2(6) | 0.0008 |
| 5^th^ finger Clinodactyly | 42(66) | 16(48) | 0.12 |
| Cleft palate | 3(5) | 6(18) | 0.058 |
| Lip abnormalities | 18(28) | 3(9) | 0.0379 |
| Camptodactyly | 12(19) | 0(0) | 0.0071 |
| Ear deformities | 31(48) | 11(33) | 0.19 |
| Congenital cardiac defects | 4(6) | 9(27) | 0.0090 |
| Speech delay | 27(42) | 11(33) | 0.51 |
| Motor delay | 10(21) | 14(42) | 0.0486 |
| Excessive sweating | 43(67) | 0(0) | <0.0001 |
| Hypoglycaemia | 14(26) | 0(0) | 0.0016 |

Underlined features are known to be associated with common imprinted causes of SRS ‘associated features’ [1,2]. ‘Other’ (not underlined) features listed are not typically associated with SRS. *NH-CSS major criteria 1, 2. Results are presented as % (number of patients).

**Supplemental Table 6** Associated and other features in Silver-Russell Syndrome patients with common imprinted causes compared to monogenic non-imprinted causes

| **Number of clinical features (%)** | **Common imprinted**  **Total n = 64** | **Non-imprinted monogenic**  **Total n = 29** | **p-value** |
| --- | --- | --- | --- |
| Placental abnormalities | 17(27) | 2(7) | 0.0493 |
| Abnormal dentition | 34(53) | 3(10) | <0.0001 |
| Micro/retrognathia | 35(55) | 6(21) | 0.0031 |
| Downturned mouth | 17(27) | 0(0) | 0.0011 |
| Delayed closure of fontanelle | 16(41) | 0(0) | <0.0001 |
| 5^th^ finger Clinodactyly | 42(66) | 7(24) | 0.0003 |
| Cleft palate | 3(5) | 0(0) | 0.54 |
| Lip abnormalities | 18(28) | 3(10) | 0.06 |
| Camptodactyly | 12(19) | 0(0) | 0.0155 |
| Ear deformities | 31(48) | 4(14) | 0.0013 |
| Congenital cardiac defects | 4(6) | 0(0) | 0.30 |
| Speech delay | 27(42) | 2(7) | 0.0006 |
| Motor delay | 10(21) | 3(10) | 0.34 |
| Excessive sweating | 43(67) | 0(0) | <0.0001 |
| Hypoglycaemia | 14(26) | 0(0) | 0.0016 |

Underlined features are known to be associated with common imprinted causes of SRS ‘associated features’ [1,2]. ‘Other’ (not underlined) features listed are not typically associated with SRS. Results are presented as % (number of patients).

**Supplemental Table 7** Associated and other features in Silver-Russell Syndrome patients with monogenic imprinted causes compared to monogenic non-imprinted causes

| **Number of clinical features (%)** | **Imprinted monogenic causes**  **Total n = 33** | **Non-imprinted monogenic**  **Total n = 29** | **p-value** |
| --- | --- | --- | --- |
| Placental abnormalities | 5(15) | 2(7) | 0.43 |
| Abnormal dentition | 3(9) | 3(10) | >0.99 |
| Micro/retrognathia | 10(30) | 6(21) | 0.56 |
| Downturned mouth | 2(6) | 0(0) | 0.49 |
| Delayed closure of fontanelle | 2(6) | 0(0) | 0.49 |
| 5^th^ finger Clinodactyly | 16(48) | 7(24) | 0.06 |
| Cleft palate | 6(18) | 0(0) | 0.0257 |
| Lip abnormalities | 3(9) | 3(10) | >0.99 |
| Camptodactyly | 0(0) | 0(0) | >0.99 |
| Ear deformities | 11(33) | 4(14) | 0.08 |
| Congenital cardiac defects | 9(27) | 0(0) | 0.0024 |
| Speech delay | 11(33) | 2(7) | 0.0132 |
| Motor delay | 14(42) | 3(10) | 0.0090 |
| Excessive sweating | 0(0) | 0(0) | >0.99 |
| Hypoglycaemia | 0(0) | 0(0) | >0.99 |

Underlined features are known to be associated with common imprinted causes of SRS ‘associated features’ [1,2]. ‘Other’ (not underlined) features listed are not typically associated with SRS. Results are presented as % (number of patients).

**Supplementary Table 8** Associated and other features seen in SRS patients with 11p15LOM compared to monogenic causes of SRS.

| **No. of clinical features (%)** | **11p15LOM** | ***CDKN1C*** | **P-value** | ***IGF2*** | **P-value** | ***HMGA2*** | **P-value** | ***PLAG1*** | **P-value** |
| --- | --- | --- | --- | --- | --- | --- | --- | --- | --- |
| Placental abnormalities | 15(34) | 0(0) | 0.0248 | 5(23) | 0.40 | 1(6) | 0.0247 | 1(9) | 0.14 |
| Abnormal dentition | 21(48) | 2(18) | 0.09 | 1(5) | 0.0003 | 2(11) | 0.0086 | 1(9) | 0.0356 |
| Micro/retrognathia | 28(64) | 0(0) | 0.0001 | 10(45) | 0.19 | 5(28) | 0.0130 | 1(9) | 0.0016 |
| Downturned mouth | 13(30) | 0(0) | 0.0497 | 2(9) | 0.07 | 0(0) | 0.0128 | 0(0) | 0.0497 |
| Delayed closure of fontanelle | 12(43) | 1(9) | 0.06 | 1(5) | 0.0028 | 0(0) | 0.0012 | 0(0) | 0.0087 |
| 5th finger Clinodactyly | 33(75) | 1(9) | 0.0001 | 15(68) | 0.57 | 5(28) | 0.0012 | 2(18) | 0.0009 |
| Cleft palate | 3(7) | 0(0) | >0.99 | 6(27) | 0.05 | 0(0) | 0.54 | 0(0) | >0.99 |
| Lip abnormalities | 12(27) | 0(0) | 0.09 | 3(14) | 0.35 | 2(11) | 0.20 | 1(9) | 0.26 |
| Camptodactyly | 7(16) | 0(0) | 0.32 | 0(0) | 0.08 | 0(0) | 0.09 | 0(0) | 0.32 |
| Ear deformities | 16(36) | 2(18) | 0.30 | 9(41) | 0.79 | 3(17) | 0.22 | 1(9) | 0.14 |
| Congenital cardiac defects | 4(9) | 0(0) | 0.57 | 9(41) | 0.0063 | 0(0) | 0.31 | 0(0) | 0.57 |
| Speech delay | 17(39) | 0(0) | 0.0119 | 11(50) | 0.43 | 0(0) | 0.0013 | 2(18) | 0.29 |
| Motor delay | 9(26) | 1(9) | 0.40 | 13(59) | 0.02 | 1(6) | 0.13 | 2(18) | 0.70 |
| Excessive sweating | 28(64) | 0(0) | 0.0001 | 0(0) | <0.0001 | 0(0) | <0.0001 | 0(0) | 0.0001 |
| Hypoglycaemia | 9(24) | 0(0) | 0.09 | 0(0) | 0.0199 | 0(0) | 0.0232 | 0(0) | 0.09 |

Underlined features are known to be associated with common imprinted causes of SRS ‘associated features’ [2,3] . ‘Other’ (not underlined) features listed are not typically associated with SRS. Abnormal dentition includes crowded/irregular teeth and small teeth. Ear abnormalities include low set, small and protruding ears. Lip abnormalities include long/smooth philtrum, thin upper lip. Congenital cardiac abnormalities include bicuspid aortic arch, total anomalous pulmonary venous return (TAPVR), ventricular and atrial septal defect. Placental abnormalities include placental hypoplasia. Lip abnormalities include long/smooth philtrum and thin upper lip.

**Supplementary Table 9** Associated and other features seen in SRS patients with upd(7)mat compared to monogenic causes of SRS.

| **No. of clinical features (%)** | **Upd(7)mat** | ***CDKN1C*** | **P-value** | ***IGF2*** | **P-value** | ***HMGA2*** | **P-value** | ***PLAG1*** | **P-value** |
| --- | --- | --- | --- | --- | --- | --- | --- | --- | --- |
| Placental abnormalities | 2(10) | 0(0) | 0.52 | 5(23) | 0.41 | 1(6) | >0.99 | 1(9) | >0.99 |
| Abnormal dentition | 13(65) | 2(18) | 0.0233 | 1(5) | <0.0001 | 2(11) | 0.0009 | 1(9) | 0.0036 |
| Micro/retrognathia | 7(35) | 0(0) | 0.0331 | 10(45) | 0.54 | 5(28) | 0.73 | 1(9) | 0.20 |
| Downturned muth | 4(20) | 0(0) | 0.26 | 2(9) | 0.40 | 0(0) | 0.10 | 0(0) | 0.26 |
| Delayed closure of fontanelle | 4(36) | 1(9) | 0.31 | 1(5) | 0.0325 | 0(0) | 0.0139 | 0(0) | 0.09 |
| 5th finger Clinodactyly | 9(45) | 1(9) | 0.055 | 15(68) | 0.21 | 5(28) | 0.32 | 2(18) | 0.24 |
| Cleft palate | 0(0) | 0(0) | >0.99 | 6(27) | 0.0216 | 0(0) | >0.99 | 0(0) | >0.99 |
| Lip abnormalities | 6(30) | 0(0) | 0.06 | 3(14) | 0.26 | 2(11) | 0.23 | 1(9) | 0.37 |
| Camptodactyly | 5(25) | 0(0) | 0.13 | 0(0) | 0.0182 | 0(0) | 0.0480 | 0(0) | 0.13 |
| Ear deformities | 15(75) | 2(18) | 0.0068 | 9(41) | 0.0334 | 3(17) | 0.0004 | 1(9) | 0.0006 |
| Congenital cardiac defects | 0(0) | 0(0) | >0.99 | 9(41) | 0.0015 | 0(0) | >0.99 | 0(0) | >0.99 |
| Speech delay | 10(50) | 0(0) | 0.0049 | 11(50) | >0.99 | 0(0) | 0.0005 | 2(18) | 0.12 |
| Motor delay | 1(7) | 1(9) | >0.99 | 13(59) | 0.0021 | 1(6) | >0.99 | 2(18) | 0.56 |
| Excessive sweating | 15(75) | 0(0) | <0.0001 | 0(0) | <0.0001 | 0(0) | <0.0001 | 0(0) | <0.0001 |
| Hypoglycaemia | 5(29) | 0(0) | 0.12 | 0(0) | 0.0107 | 0(0) | 0.0191 | 0(0) | 0.12 |

Underlined features are known to be associated with common imprinted causes of SRS ‘associated features’ [2,3]. ‘Other’ (not underlined) features listed are not typically associated with SRS. Abnormal dentition includes crowded/irregular teeth and small teeth. Ear abnormalities include low set, small and protruding ears. Lip abnormalities include long/smooth philtrum, thin upper lip. Congenital cardiac abnormalities include bicuspid aortic arch, total anomalous pulmonary venous return (TAPVR), ventricular and atrial septal defect. Placental abnormalities include placental hypoplasia. Lip abnormalities include long/smooth philtrum and thin upper lip.

**References**

1. Wakeling EL, Brioude F, Lokulo-Sodipe O, O’Connell SM, Salem J, Bliek J, et al. Diagnosis and management of Silver–Russell syndrome: first international consensus statement. Nat Rev Endocrinol. 2017;13:105–24.

2. Kurup U, Lim DBN, Palau H, Maharaj A V, Ishida M, Davies JH, et al. Approach to the Patient With Suspected Silver-Russell Syndrome. J Clin Endocrinol Metab. 2024;

3. Wakeling EL, Amero SA, Alders M, Bliek J, Forsythe E, Kumar S, et al. Epigenotype-phenotype correlations in Silver-Russell syndrome. J Med Genet. 2010;47:760–8.
